# Clinical efficacy and radiographic K-rod stabilization for the treatment of multilevel degenerative lumbar spinal stenosis

**DOI:** 10.1186/s12891-020-03466-0

**Published:** 2020-07-06

**Authors:** Chaohua Fu, Tianjun Chen, Yuhao Yang, Hua Yang, Maohui Diao, Guowei Zhang, Zhisheng Ji, Hongsheng Lin

**Affiliations:** 1 Department of Orthopedics, the First Affiliated Hospital of Jinan University, Guangzhou, 510630 Guangdong China; 2 Department of Orthopedics, the Jiangmen hospital of Sun Yat-Sen University, Jiangmen, China; 3 Department of Orthopedics, Shenzhen Baoan Second People’s hospital, Shenzhen, China

**Keywords:** K-rod, Dynamic stabilization system, Multisegmental degenerative lumbar spinal stenosis, Selective fusion

## Abstract

**Background:**

This study compares the use of radiographic K-Rod dynamic stabilization to the rigid system for the treatment of multisegmental degenerative lumbar spinal stenosis (MDLSS).

**Methods:**

A total of 40 patients with MDLSS who underwent surgical treatment using the K-Rod (*n* = 25) and rigid systems (*n* = 15) from March 2013 to March 2017 were assessed. The mean follow-up period was 29.1 months. JOA, ODI, VAS and modified Macnab were assessed. Radiographic evaluations included lumbar lordosis angle, ISR value, operative and proximal adjacent ROM. Changes in intervertebral disc signal were classified according to Pfirrmann grade and UCLA system.

**Results:**

JOA, ODI and VAS changed significantly after the operation to comparable levels between the groups. However, the lumbar lordosis significantly decreased at final follow-up between both groups. The ROM of the proximal adjacent segment increased at final follow-up, but the number of fixed segment ROMs in the K-Rod group were significantly lower at the final follow-up than observed prior to the operation. In both groups, the ISR of the proximal adjacent segment decreased, most notably in the rigid group. The ISR of the non-fusion fixed segments in the K-Rod group increased post-operation and during final follow-up. The levels of adjacent segment degeneration were higher in the rigid group vs. the K-Rod group according to modified Pfirrmann grading and the UCLA system.

**Conclusions:**

Compared with the rigid system for treatment of MDLSS, dynamic K-Rod stabilization achieves improved radiographic outcomes and improves the mobility of the stabilized segments, minimizing the influence on the proximal adjacent segment.

## Background

Degenerative lumbar spinal stenosis (DLSS) refers to a range of degenerative factors due to bone or fibrous structural volume and morphological abnormalities, resulting in nerve roots, cauda equina compression and clinical symptoms of a class of diseases [1–3]. With the aging of society, DLSS is increasing in prevalence [4]. Furthermore, intervertebral disc degeneration in elderly individuals is not limited to a single segment, but occurs due to multi-segmental interactions. Multisegmental degenerative lumbar spinal stenosis (MDLSS) often seriously affects the normal life of the elderly, and sometimes they need surgical treatment [5, 6]. The use posterior lumbar interbody fusion (PLIF) was the front-line therapy for DLSS [7], but long segmental spinal fusion surgery often brings many complications to patients, including donor place ailment, morbidity of the surgery, and adjacent segment disease [8, 9].

Based on the common complications after spinal fusion, an ideal internal fixation system is designed to stabilize the spine while allowing the fixation site to share load and transmit stress more effectively, avoiding stress concentration and reducing stress shielding. With the development of new spinal surgery concepts, non-fusion internal fixation systems have been proposed [10, 11], which can be roughly divided into three categories: (a) pedicle screw-ligaments; (b) semi-rigid fixation of the lumbar spine; (c) dynamic pedicle screws. Dynamic K-Rod fixation employs titanium alloy pedicle screws, alloy cable rods, and polyaryletherketone (PEEK) spacers [12]. This system is designed to establish a consistent range of motion (ROM) in the fixed segment, maintaining spinal stability and preventing adjacent segment degeneration (ASD) [12, 13]. As we know, non-fusion fixation of the spinal column can protect the degeneration of adjacent segments of the spinal column [14]. Even more, the K-Rod dynamic stabilization fixation system can perform selective fusion of spinal segments, which means it can fix both the fusion segment and the non-fusion segment of the spine [15]. If a segment needs to be fused and fixed, a metal ring can be applied to the K-Rod connecting rod, which makes K-rod with dynamic function lose elasticity and become rigid fixation.

To date, clinical studies on the dynamic K-Rod system for the treatment of DLSS have not been performed [16]. Here, we retrospectively analyzed the efficacy of dynamic K-Rod stabilization for the treatment of DLSS.

## Methods

### General data

From March 2013 to March 2017, 40 DLSS patients admitted to our institute were included. Among them, 25 (12 males and 13 females) underwent dynamic K-Rod stabilization, while 15 (8 males and 7 females) underwent rigid internal fixation. Patients were followed up ≥12 months and were assessed by MRI and X-rays (Table 1).

**Table 1:** General data

General data	K-Rod(*N* = 25)	Rigid(*N* = 15)	*P*
Age (years)	66 ± 6.79	61 ± 7.17	0.674
SEX			
Female	12 (48%)	8 (53.33%)	0.752
Male	13 (52%)	7 (46.66%)	
Follow-up (months)	29.48 ± 4. 97	28.26 ± 5. 88	0.483
Operation Time (mins)	289 ± 34.40	307 ± 49.06	0.162
Intraoperative blood loss (mL)	480 ± 270.03	462 ± 294.14	0.845
Postoperative drainage (mL)	340 ± 120.34	332 ± 55.32	0.810
Hospital stay (days)	15 ± 3.50	16 ± 3.35	0.748
Expenses (ten thousands)	11.7 ± 1.29	10.6 ± 1.64	0.018

Note: Data are presented as mean ± standard deviation. *P* values are based on the t test; *P* > 0.05 compared with K-Rod and Rigid

### Inclusion, exclusion and fusion criteria

Inclusive criteria: (a) spinal surgery patients admitted from March 2013 to March 2017; (b) diagnosis of lumbar spinal stenosis, conservative treatment for more than 3 months being ineffective, two or more surgical lumbar segments; (c) good compliance, informed consent to the surgical program, actively co-operating with the treatment of clinical researchers; (d) The patients were followed up for more than 1 year. Exclusive criteria: (a) non-multilevel degenerative lumbar spinal stenosis, such as traumatic lumbar spondylolisthesis, tumor, spinal tuberculosis, lumbar spine fracture; (b) severe osteoporosis, severe scoliosis; (c) past history of lumbar surgery; (d) poor physical condition or unable to tolerate surgery. Those with contraindications were: (e) incomplete medical records or imaging data. Fusion criteria [17]: (a) severe disc degeneration, (b) intervertebral instability, (c) significant lumbar degenerative scoliosis, kyphosis or spondylolisthesis, (d) bilateral facetectomy > 1/3–1/2, excision ≥50% of the pars interarticularis, with bilateral discectomy performed in addition to partial facetectomy.

### Operative methods and postoperative management

The same surgical team performed all operative methods at our institute. Posterior spine surgeries were performed in the prone position. A midline incision and subperiosteal dissection of the erector spine muscles was performed. Segments were then exposed and pedicle screws were inserted under X-ray guidance. Dependent on the condition of the patient, interlaminar decompression and laminotomy were performed with destruction of the facet joints carefully prevented. Next, removal of the intervertebral disk was performed and the interbody fusion cage was filled with bone and inserted. The selection of fusion segments was based upon disease severity and level of disc degeneration in the K-rod group. For the rigid group, all segments were fused. In the rigid group, rigid titanium rods were placed at the end of the nail, but in the K-Rod group, longitudinal connecting rods were assembled according to whether fusion was needed, a semi-hollow rod was inserted at the fusion segment and a PEEK rod was inserted at the non-fusion segment. At the end of the procedure, the incision was fully flushed and drained. The deep fascia, subcutaneous and skin were sutured layer by layer.

After the operation, the patients were given broad-spectrum antibiotics one to three days after surgery. When the drainage volume declined to ≤50 mL over the 24 h period, the tube was removed. Upon discharge, patients were regularly re-examined, and wore waist circumference protection for 3 months. Excessive weight-bearing activities were prohibited for 6 months.

### Radiological assessments

We performed all patient assessments immediately after the operation, after 3 months, and at final follow-up. We used JOA, VAS and ODI scores to assess lower back pain and quality of life. The clinical efficacy of the two groups was evaluated by modified Macnab criteria.

Radiological measurements were performed as follows: (a) the fusion rate was established according to the judgment standard of bone fusion segment by Suk [18]; (b) lumbar lordosis angle: the angle between the L1 vertebral superior endplate extension line and the S1 vertebral superior endplate extension line; (c) segmental ROM was assessed from the angle of the inferior surface of the upper vertebrae to the superior surface of the lower vertebrae through lateral standing lumbar flexion-extension X-rays; (d) ISR was measured from the ventral intervertebral space height (VH), dorsal intervertebral space height (DH), and the VH of the upper adjacent vertebral body (UVH): ISR = (VH + DH)/2UVH; (e) patients received lumbar MRI to assess any changes in height of the adjacent degenerative intervertebral discs. T2-weighted sagittal and axial MRI was performed to assess the levels of disc degeneration according to Pfirrmann’s methods; (f) UCLA system: the degree of intervertebral space degeneration was evaluated by X-ray. Radiographs were assessed in triplicate by two experienced spine surgeons and the means of each parameter recorded. Disagreements were overcome through discussion or consultation.

### Statistics

Clinical data and imaging measurements of the patients were analyzed on SPSS19.0 shown as the mean ± SD. Enumeration data were compared through the Chi square test. Comparison of the categorical data was performed using a Wilcoxon signed rank test. *P*-values < 0.05 showed significant differences.

## Results

### Patient baseline characteristics

Follow-ups were performed at 12–36 months; the K-Rod group had an average of 29.5 months, and the rigid group had 28.3 months. Age, bleeding volume during operation, follow-up date, total drainage volume, total hospitalization time and operation time did not significantly differ between the groups. The total cost of hospitalization in K-Rod group was ¥11.7 ± 1.29 ten thousand, and that in Rigid group was 10.6 ± 1.64 ten thousand. The costs of the K-Rod group exceeded those of the rigid fixation group (*P* < 0.05) (Table 1).

### Clinical efficacy

The scores of JOA, ODI and VAS were compared between K-Rod group and rigid group. The *P* values of JOA, ODI and VAS in K-Rod group were less than 0.05 before and after the operation. Improvements in JOA, ODI and VAS were comparable between the groups (Table 2).

**Table 2:** Scores of JOA, ODI and VAS

Index	K-Rod(N = 25)			Rigid(N = 15)		
	JOA	ODI	VAS	JOA	ODI	VAS
Pre-operative	14.6 ± 1.26	37.92 ± 2.58	6.48 ± 0.96	14.6 ± 0.828	38.00 ± 2.20	6.53 ± 0.92
Post-operative	22.68 ± 0.85	15.24 ± 2.71	2.24 ± 0.723	22.40 ± 0.986	15.20 ± 1.01	1.52 ± 1.01
*P*	0.000	0.000	0.000	0.000	0.000	0.000
*P*′	JOA (0.461)		ODI (0.247)		VAS (0.391)	

Note: Data are the mean ± SD. Data were compared through t-tests, *P* means Post- vs. pre-operative, *P* < 0.05; *P*′ means K-Rod group compare with rigid group, *P* > 0.05. *P* < 0.05 mean statistically significant differences

The clinical efficacy of modified Macnab in K-Rod group was 84.00%. K-Rod caused no improvement over the rigid group (*P* > 0.05) (Table 3).

**Table 3:** Clinical assessment of modified Macnab

Group	N	Excellent	Good	Fair	Poor	The Excellent/Good rate	*P*
K-Rod	25	6	15	3	1	84.00%	1.0
Rigid	15	4	8	2	1	80.00%	

Note: Data were compared using a chi-square test, *P* < 0.05 significant difference

### Radiologic outcomes of fusion rate

At the last follow-up, there were 17 fusion segments in the K-Rod group; 16 were judged as strong fusion, and 1 was judged as possible fusion, with a fusion rate of 94.11%. There were 37 fusion segments in the rigid fixation group, 36 were judged as strong fusion, and 1 was judged as possible fusion. The fusion rate was 97.30%, which was comparable across the groups (*P* > 0.05) (Table 4).

**Table 4:** Fusion rates of the two groups

Grading	K-Rod (*N* = 17)	Rigid (*N* = 37)	*P*
Fusion	16	36	
Possible fusion	1	1	
Non-fusion	0	0	
Fusion rate (%)	94.11%	97.30%	0.535

Note: *P* values are based on the chi-square test, *P* < 0.05 mean statistically significant differences

### ROM and lumbar lordosis angle assessments

**Fig. 1:**
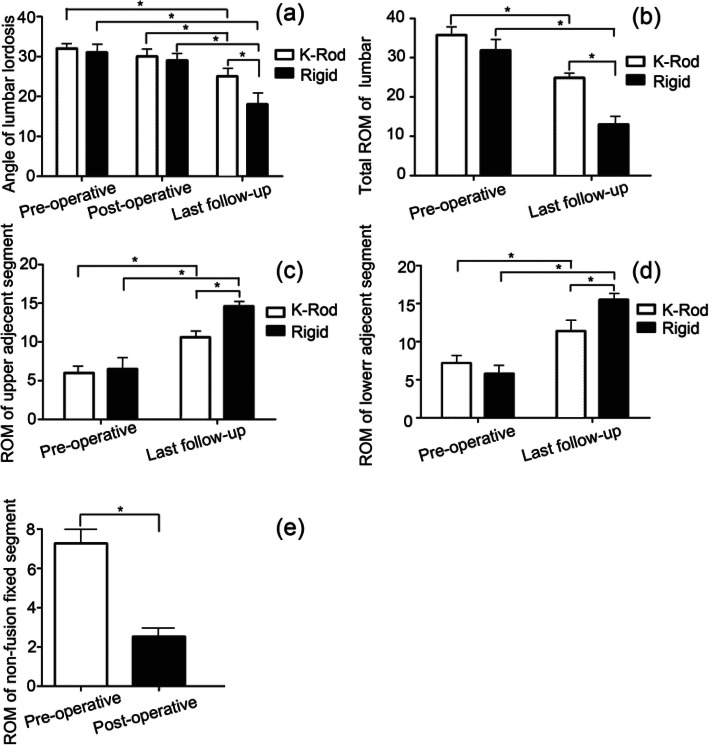
Effects of dynamic vs rigid K-Rod stabilization on the lumbar lordosis angle and motion during DLSS treatment. **a** Lumbar lordosis angle between K-Rod and rigid groups; **b** the total lumbar ROM of the two groups at the last follow-up; **c** & **d** ROM of the lower and upper adjacent segments at final follow-up; **e** ROMs of non-fusion fixed segments pre-operatively and post-operatively

Preoperative and postoperative radiologic parameters including lumbar lordosis angle and ROM in the K-Rod and rigid groups are shown in Fig. 1. The lumbar lordosis angle between the two groups was similar in both pre-operation and post-operation (*P* > 0.05). However, the lumbar lordosis angle was lower in the K-Rod group at the last follow-up (*P* < 0.05), but not post-operation (*P* > 0.05). The decrease of the K-Rod group was slower than that of the rigid group, suggesting that the K-Rod group had a positive effect on lumbar lordosis angle maintenance (Fig. 1a). Furthermore, the total lumbar ROM of both groups at the final follow-up was lower than prior to the operation (*P* < 0.05) (Fig. 1b); the lower and upper adjacent showed an improved and significantly increased ROM (*P* < 0.05), while the ROM of the adjacent segments in the rigid group (13.82 ± 6.65)° increased vs. the K-Rod group (24.94 ± 9.61)° at the last follow-up (*P* < 0.05). Thus, the K-Rod group led to a higher ROM of the lumbar spine (*P* < 0.05). The upper and lower adjacent segments did not differ between groups (*P* > 0.05) (Fig. 1c, d). At the last follow-up, the number of fixed segment ROMs were lower in the K-Rod group compared with those prior to the operation (*P* < 0.05), suggesting that fixed segment ROMs in the K-Rod group were limited and that the K-Rod dynamic stabilization system can effectively stabilize non-fusion intervertebral activity (Fig. 1e).

### Radiologic outcomes of ISR value

**Fig. 2:**
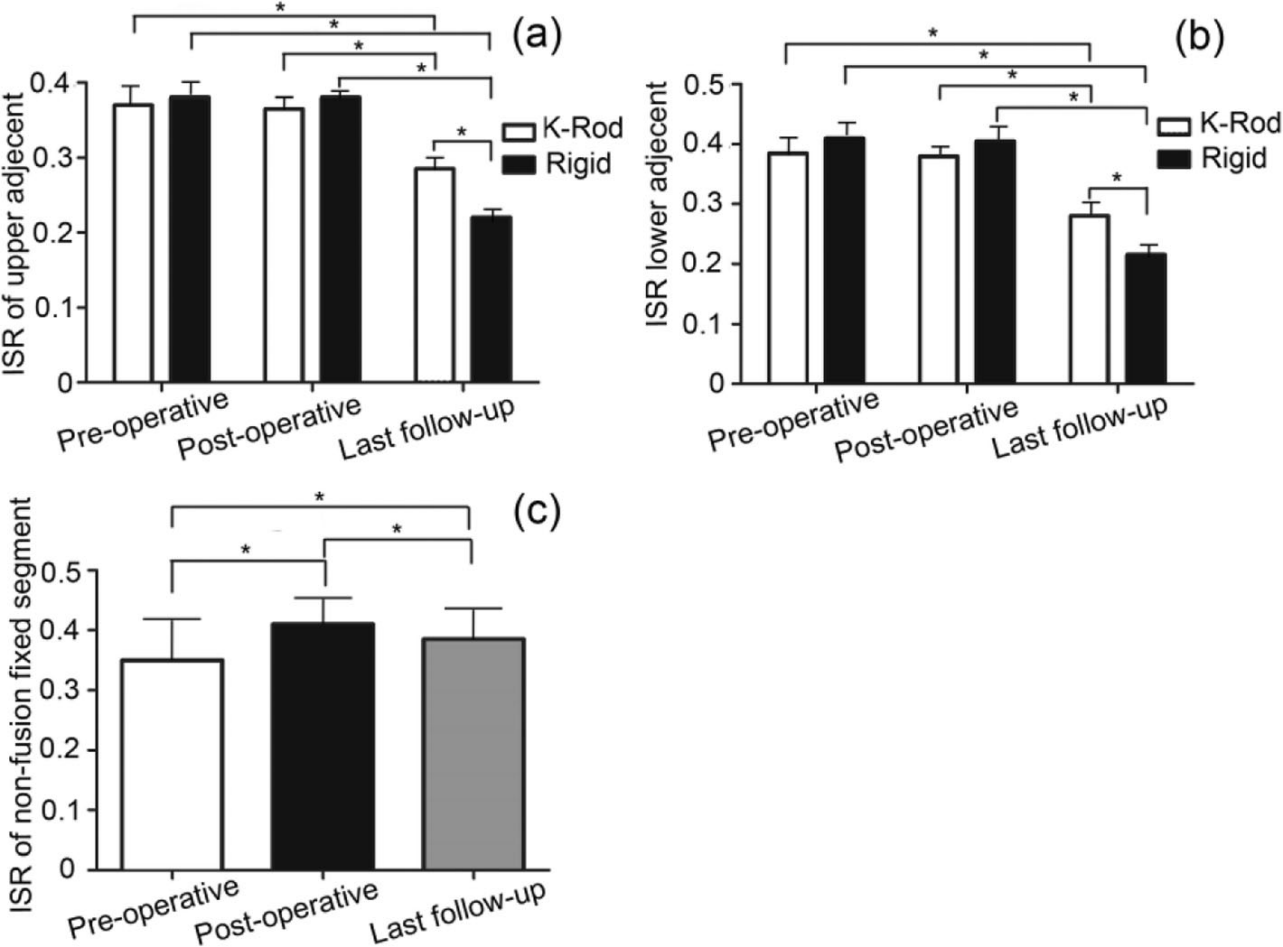
Comparison of dynamic and rigid K-Rod stabilization on lumbar ISR and intervertebral disc degeneration in the treatment of MDLSS. **a** The ISR value of upper adjacent segment in K-Rod and rigid groups at different point in time including pre-operative, post-operative and last follow up; **b** the ISR value of lower adjacent segment in the K-Rod and rigid groups at different points in time including pre-operative, post-operative, and last follow-up; (**b**) ROM of lower and upper segments at final follow-up; **c** ISR value of non-fusion fixed segment at pre-operative, post-operative, and last follow-up

ISR values in the K-Rod and rigid groups are shown in Fig. 2. The ISR value of upper adjacent segment between the two groups was similar including pre-operation and post-operation (*P* > 0.05). However, the ISR values of the upper adjacent segments were higher in the K-Rod group at the last follow-up (*P* < 0.05), suggesting that K-Rod prevented the degeneration of adjacent segments (Fig. 2a). The results of lower adjacent segments were quite similar to upper adjacent segments, which confirms the results again (Fig. 2b). The ISR values of the non-fusion and fixed segments in the K-Rod group increased after operation (*P* < 0.05), but declined significantly until the last follow up (*P* < 0.05). The ISR value of the last follow-up in the K-Rod group exceeded that observed prior to the operation (*P* < 0.05) (Fig. 2c).

### Radiologic outcomes of degeneration in adjacent segments

According to the modified Pfirrmann grading system, the incidence of degeneration of adjacent segments was 2.5% in the K-Rod group and 26.3% in the rigid group. The incidence of adjacent segment degeneration in the K-Rod group was significantly lower than the rigid group (*P* < 0.05) (Table 5).

**Table 5:** The modified Prirrmann grade rate of proximal adjacent in two groups

	Proximal adjacent segment	P
K-Rod	2.5%	0.012
Rigid	26.3%	

Note: Data were compared using a chi-square test, *P* < 0.05 mean statistically significant differences

According to the UCLA system, the incidence of adjacent segment degeneration was 5.0% in the K-Rod group and 31.6% in the rigid group. Adjacent segment degeneration of the K-Rod group was significantly lower than the rigid group (*P* < 0.05) (Table 6).

**Table 6:** UCLA system evaluation of intervertebral space

	Proximal adjacent segment	*P*
K-Rod	5.0%	0.018
Rigid	31.6%	

Note: Data were compared using a chi-square test, *P* < 0.05 mean statistical significant differences

### Radiologic outcomes of typical case

**Fig. 3:**
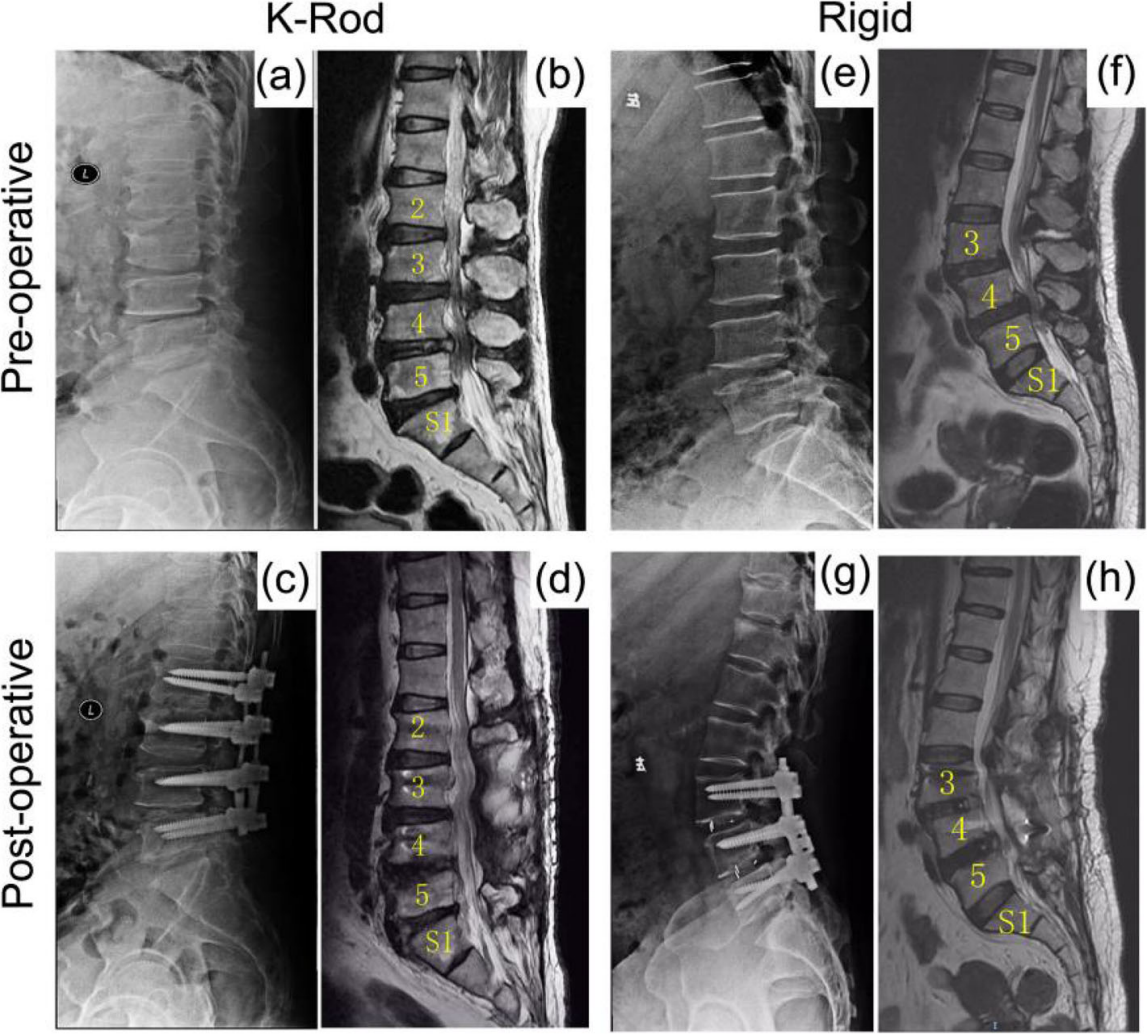
Comparison of pre-operative and post-operative imaging: **a-d**: K-rod; **e-g** rigid (DR: **a, c, e** and **g**; MRI: **b, d, f** & **h**); **a**, **b**, **e** and **f**: pre-operative images; **c**, **d**, **g** & **h**: post-operative images of patients

Typical case (Fig. 3): Fig. 3a-d (K-rod group): Male, 63 years old, with DLSS (L2/3, L3/4, and L4/5), K-Rod dynamic internal fixation and fusion, postoperative symptoms significantly improved. According to the improved Pfirrmann classification, adjacent segment L1/2 were all grade 3, adjacent segment L5/S1 were all grade 6, no significant degeneration. Figure 3e-h (Rigid group): A 61-year-old female patient with DLSS (L3/4, L4/5) underwent L3/4, L4/5 decompression and rigid fixation. Postoperative symptoms improved significantly. According to the modified Pfirrmann classification, L2/3 of the upper adjacent segment degenerated from preoperative grade 4 to postoperative grade 5, and L5/S1 of the lower adjacent segment degenerated from preoperative grade 3 to postoperative grade 4.

## Discussion

K-Rod dynamic stabilization preserves the functionality of fixed segments, thus maintaining the stability of the spine and avoiding ASD [12, 13, 16]. When the activity of fixed segments and lumbar lordosis are maintained, dynamic K-Rod stabilization prevents the incorrect motion of unstable segments and reduces intervertebral disc loads and facet joints, thus promoting recovery [16, 19]. In contrast to the rigid system, K-Rod dynamic technology circumvents the stress caused by movements of the adjacent segments, preventing ASD. Theoretically, the dynamic K-Rod system can outperform typical rigid systems.

Both strategies prevent DLSS and provide beneficial short-terms effects [16]. We observed improvements in JOA, VAS and ODI post-operatively and at final follow-up, but differences between the K-Rod and rigid groups were not significant. In addition, the clinical efficacy of modified Macnab in the two groups was similar. Therefore, compared with the rigid group, the K-Rod group achieved a comparable outcome to traditional pedicle screws in terms of short-term benefits. This highlighted the beneficial effects of the dynamic K-Rod system for DLSS therapy.

The dynamic system is designed to reduce the pressure on the intervertebral joint and reduce its compensatory activity, and dynamic stabilization may indirectly reduce the adjacent segment degeneration [20]. In DLSS patients, destabilization promotes ASD development and is an area of intense research interest [21]. In this study, lumbar total ROM of the K-Rod group during the follow-up exceeded that of the rigid group, indicating that K-Rod can retain a certain degree of lumbar total ROM. For adjacent segment ROMs, the upper and lower adjacent segment ROMs in the K-Rod group changed less than those in the rigid group. It can be seen that K-Rod dynamic stabilization system can have little effect on the relative activity of adjacent segments. Postoperative fixation segmental mobility of the K-Rod group was significantly lower than pre-operation, but part of the activity was retained. This shows that the K-Rod system has a certain degree of stabilization on the lumbar surgical segment, but it is different from the rigid fusion segmental lack of intervertebral mobility [22, 23] . In summary, the dynamic K-Rod system in part improved/preserved the ROM of surgical segments through its ability to promote stabilization. ROM of the lumbar spine similarly improves.

The lumbar disc height of proximal adjacent segments prior to- and post-treatment were comparable, suggesting that dynamic K-Rod stabilization reduces the influence of the intervertebral space. The intervertebral space recovers after the operation, but does decrease following long-term follow-up [24, 25]. The possible reason is that the intervertebral space will be properly elevated during lumbar surgery, so the value of ISR post-operation is slightly higher than that of preoperative ISR. In the K-Rod group, the intervertebral space was higher pre-operation and maintained through K-Rod mediated fixation. However, with the occurrence of intervertebral space degeneration, the height of the intervertebral space was gradually lost in the K-Rod group. In addition, the ISR values of adjacent segments in the rigid group decreased more rapidly than in the K-Rod group, indicating that the K-Rod dynamic stabilization system could slow down the degeneration of adjacent segments. The lumbar lordosis angle between the two groups was similar in both pre-operation and post-operation. However, the lumbar lordosis angle was lower in the K-Rod group at the last follow-up. It suggested that the K-Rod group had a positive effect on lumbar lordosis angle maintenance and it may lead to better treatment effect.

Compared with rigid fixation system, K-Rod stabilization reduced the incidence of adjacent segment degeneration. In this study, the incidence of degeneration of adjacent segments was 2.5% in the K-Rod group and 26.3% in the rigid group according to the modified Pfirrmann grading system. Additionally, according to the UCLA system, the incidence of adjacent segment degeneration for K-Rod was 5.0% vs. 31.6% in the rigid group. K-Rod led to a lower incidence of adjacent segment degeneration compared with the rigid group. These results indicate that the K-Rod dynamic stabilization system can slow the degeneration of adjacent segments to some extent.

Upon comparison of lumbar discectomy and interbody fusion, K-Rod dynamic stabilization could remove the herniated nucleus pulposus and relieve compression of the nerves. The lumbar disc height could also be recovered during operation to maintain the structure of the lumbar spine. The ROM of the operative segment was also preserved, which reduced stress on the segments, and provided a compensation of the ROM [21, 26]. Dynamic K-Rod stabilization delays the occurrence of adjacent-level degeneration [11, 13, 16]. Due to the relatively short follow-up time of this study, it will take longer and more follow-up to further confirm the reliability of the results.

## Conclusions

Compared with the rigid system for treatment of MDLSS, K-Rod dynamic stabilization system improves radiographic outcomes and ROM of the stabilized segments, with a reduced influence on the proximal adjacent segment through the preservation of segmental motion and intervertebral height. This maintains lumbar lordosis, and reduces the degeneration of adjacent segments.

## Data Availability

The datasets used and analyzed during the current study are available from the corresponding author on reasonable request.

## Abbreviations

ASD: Adjacent segment degeneration
DH: Dorsal intervertebral space height
DLSS: Degenerative lumbar spinal stenosis
MDLSS: Multisegmental degenerative lumbar spinal stenosis
PLIF: Posterior lumbar interbody fusion
PEEK: Polyaryletherketone
ROM: Consistent range of motion
VH: Ventral intervertebral space height
